# Effects of Recombinant *Toxoplasma gondii* Citrate Synthase I on the Cellular Functions of Murine Macrophages *In vitro*

**DOI:** 10.3389/fmicb.2017.01376

**Published:** 2017-07-21

**Authors:** Xinchao Liu, Qunshan Ma, Xiaoni Sun, Mingmin Lu, Muhammad Ehsan, Muhammad Waqqas Hasan, Lixin Xu, RuoFeng Yan, XiaoKai Song, XiangRui Li

**Affiliations:** Preventive Veterinary Medicine Department, College of Veterinary Medicine, Nanjing Agricultural University Nanjing, China

**Keywords:** *Toxoplasma gondii*, citrate synthase I, cellular, functions, murine macrophages

## Abstract

Toxoplasmosis, which is one of the most widespread zoonoses worldwide, has a high incidence and infection can result in severe disease in humans and livestock. Citrate synthase (CS) is a component of nearly all living cells that plays a vital role in the citric acid cycle, which is the central metabolic pathway of aerobic organisms. In the present study, the citrate synthase I gene of *Toxoplasma gondii (T. gondii)* (TgCSI) was cloned and characterized. The TgCSI gene had an open reading frame of 1665 bp nucleotides encoding a 555 amino acid protein with a molecular weight of 60 kDa. Using western blotting assay, the recombinant protein was successfully recognized by the sera of rats experimentally infected with *T. gondii*, while the native protein in the *T. gondii* tachyzoites was detected in sera from rats immunized with the recombinant protein of TgCSI. Binding of the protein to murine macrophages was confirmed by immuno fluorescence assay. Following incubation of macrophages with rTgCSI, the rTgCSI protein was found to have a dual function, with low concentrations (5–10 μg/mL) enhancing phagocytosis and high levels (80 μg/mL) inhibiting phagocytosis. Investigation of murine macrophage apoptosis illustrated that 5 μg/mL rTgCSI protein can significantly induce early apoptosis and late stage apoptosis (^*^p < 0.05), while 10 μg/mL rTgCSI protein significantly induced early apoptosis, but had no effect on late stage of apoptosis (^**^p < 0.01), and 80 μg/mL rTgCSI protein inhibited late stage apoptosis of macrophages (^*^p < 0.05). Cytokine detection revealed that the secretion of interleukin-10, interleukin-1β, transforming growth factor-β1 and tumor necrosis factor-α of macrophages increased after the cells were incubated with all concentration of rTgCSI, with the exception that 5 μg/mL rTgCSI had no effect on the secretion of interleukin-10 and interleukin-1β. However, secretion of NO and cell proliferation of the macrophages were substantially reduced. Taken together, these results suggested that TgCSI can affect the immune functions of murine macrophages by binding to the cells *in vitro*.

## Introduction

*Toxoplasma gondii* is a ubiquitous protozoan parasite thought to infect about one third of the world's human population. The organism is able to infect all warm-blooded animals, which makes it a significant zoonotic and veterinary pathogen (Kim and Weiss, 2008). In immunocompromised patients, such as those with HIV/AIDS, infection can cause life-threatening encephalitis and be fatal if not recognized and treated soon after infection (Montoya and Liesenfeld, 2004). Infection with *T. gondii* in animals has resulted in economic losses to livestock or animal husbandry (Caldeira et al., 2011; Valença et al., 2011). Infection occurs through ingestion of food or water contaminated with oocysts and eating undercooked or raw meat containing tissue cysts (Hill and Dubey, 2013). *Toxoplasma gondii* is an obligate intracellular parasite that can infect almost all cell types. The parasitophorous vacuole of *T. gondii* is a specialized compartment for replicating in the cytoplasm (Miller et al., 2003).

Macrophages play an important role in both innate and adaptive immune responses. Some of the classical macrophage functions include phagocytosis, the killing of pathogens and tumor cells and cytokine production. Following infection by *T. gondii*, macrophages are important effector cells to control and kill toxoplasma, but they also serve as a host cell for *T. gondii* survival and proliferation (Zhou et al., 2011). Therefore, the effects of *T. gondii* protein on macrophages *in vitro* might reflect how the protein works in infected hosts.

Oxidative phosphorylation is a canonical function of eukaryotic mitochondria. The citric acid cycle, which is also known as the tricarboxylic acid (TCA) cycle or the Krebs cycle, is a series of chemical reactions used by all aerobic organisms to generate energy through the oxidation of acetyl-CoA derived from carbohydrates, fats and proteins into carbon dioxide and chemical energy in the form of adenosine triphosphate (Kay and Weitzman, 1987). A functional respiratory chain and oxidative phosphorylation have been confirmed to exist in the mitochondrion of tachyzoites (Vercesi et al., 1998). Citrate synthase is a central enzyme of multiple important metabolic pathways in cells. Citrate synthase catalyzes the synthesis of citrate from oxaloacetate and acetyl-CoA. Depending on the location of citrate synthase in eukaryotes, it is divided into mitochondrial citrate synthase, peroxisome citrate synthase and glyoxysome citrate synthase (Schnarrenberger and Martin, 2002). Fleige (Fleige et al., 2008) reported that there were also three kinds of citrate synthase in the *T. gondii* TCA cycle called CSI, CSII, and CSIII, among which CSI is located in the mitochondrion. Another report has shown that the rate of oxidation of TCA cycle intermediates decreases when CSI is absent (Kispal et al., 1988). However, the relative contribution of CSI to the immune response in infected hosts is still unclear.

Consequently, in this study, we selected murine macrophages to investigate the functions of CSI recombinant protein of *T. gondii* on the cellular level *in vitro*.

## Materials and methods

### Ethics statement

This study was conducted in accordance with the recommendations of the guidelines of the Animal Ethics Committee, Nanjing Agricultural University, China. The protocol was approved by the Science and Technology Agency of Jiangsu Province (approval ID, SYXK (SU) 2010–0005).

### Animals

Eight week old female Sprague Dawley (SD) rats were bought from the Centre of Comparative Medicine, Yangzhou University (Yangzhou, China) and maintained under specific pathogen free conditions.

### Parasites and cell culture

*Toxoplasma gondii* RH strain (Type I) was preserved in liquid nitrogen at the Laboratory of Veterinary Molecular and Immunological Parasitology, Nanjing Agricultural University, China. Parasites were maintained *in vitro* on Vero cells in Dulbecco's modified Eagle's medium (Gibco, Beijing, China) supplemented with 10% fetal bovine serum (Gibco, USA) at 56°C and 1% Penicillin-Streptomycin (Gibco, Beijing, China).

Murine macrophages (Ana-1) were grown and maintained in the same medium as Vero cells. Medium was changed every 3 days. Cells between passage 3 and 9 were used for all experiments.

### Obtaining recombinant *T. gondii* CSI protein

#### Total RNA extraction and reverse transcription of T. gondii

Total RNA was isolated from *T. gondii* tachyzoites using an E.Z.N.A. Total RNA Kit (OMEGA) according to the manufacturer's instructions. The quantity of RNA was estimated by measuring the optical density at 260 nm (OD260) using a spectrophotometer (BioRad Laboratories, Hercules, CA, USA). RNA with an absorption ratio (OD260/OD280) between 1.8 and 2 was used for reverse transcription according to the manufacturer's instructions (TaKaRa Biotechnology).

#### Cloning, expression, and purification of TgCSI

The entire ORF of the *T. gondii* CSI gene was amplified from tachyzoites cDNA by PCR with the following primers containing restriction enzyme sites (underlined) and protective bases fused to the 5′ end (*Bam*)H I forward primer, 5′-CGCGGATCCATGCTCCTCTCACGCCTACGC-3′, *Xho* I reverse primer, 5′-CCGCTCGAGCTAGTTGCCTTTCCTCTCAACGCA-3′). The products were cloned into pMD18-T vector (TaKaRa Biotechnology) and transformed into *E. coli* (DH5α) competent cells. The prokaryotic expression vector pET-32a (+)-TgCSI was constructed and transformed into *E. coli* (BL21). The recombinant TgCSI protein fused with a polyhis-tag was expressed after induction with 1 mM Isopropyl-b-D-thiogalactopyranoside (IPTG, Sigma-Aldrich, USA). Finally, recombinant TgCSI protein was purified using a Ni^2+^-nitrilotriacetic acid (Ni-NTA) column (GE Healthcare, USA) according to the manufacturer's instructions (Zhang et al., 2014, 2016). Furthermore, the fusion protein encoded by the polyhis-tag of pET-32a (+) vector (pET-32a protein) was expressed.

All oligonucleotides used in this study were synthesized by Invitrogen Biotechnology Co. Ltd. (Shanghai, PR China).

#### Producing antisera against rTgCSI protein and T. gondii

Polyclonal antibodies against rTgCSI were produced as described by Yanming Sun (Yanming et al., 2007). Briefly, two rats were immunized with 300 μg rTgCSI protein emulsified with complete Freund's adjuvant (Sigma, USA) and treated subcutaneously. Two weeks later, a booster dose using rTgCSI protein mixed with Freund's incomplete adjuvant was administered to the rats. Another three boosters were administered to rats at 1-week intervals. One week after the last injection, sera were obtained. To produce rat antisera against *T. gondii*, two SD rats were infected with *T. gondii* experimentally, and the sera were harvested 3 weeks post-infection.

#### Western blotting analysis for recombinant and native CSI

*Toxoplasma gondii* tachyzoites was sonicated on ice using the sonication system pulse for 5 s on and 10 s off for 50 cycle times. Then spin tubes at 4°C in a microcentrifuge at maximum speed for 10 min. The condensed supernatant was used as the total soluble protein of *T. gondii* tachyzoites.

Samples including the total soluble protein of *T. gondii* tachyzoites and the recombinant protein of CSI were separated by reducing SDS-PAGE. The proteins were then transferred to nitrocellulose membrane (Millipore, USA). After being blocked with 5% (w/v) skimmed milk powder in TBS (Tris-buffer saline)-Tween20 (TBST), the membranes were incubated with primary antibody (respective rat antisera) for 2 h at 37°C (1:100 dilutions). The membranes were subsequently washed three times and incubated with horseradish peroxidase (HRP)-conjugated goat anti-rat IgG (Sigma, USA) at 37°C for 1 h. Finally, the bound antibody was identified using a DAB Horseradish Peroxidase Color Development Kit (Beyotime Biotechnology).

### Ability of rTgCSI to bind to murine macrophages (Ana-1)

A total of 5 × 10^5^ Ana-1 cells were inoculated in a 12-well plate (Corning Costar, NY, USA). Next, 20 μg/mL of rTgCSI protein and pET-32a protein and an equal volume of PBS as a negative control were added to each well. The mixtures were then incubated at 37°C for 1 h and washed three times with PBS. Next, Ana-1 cells were fixed with 4% phosphate-buffered (PBS) paraformaldehyde for 30 min, washed three times in PBST containing 0.05% Tween-20 (PBST), and blocked with PBST containing 5% (w/v) BSA for 1.5 h at 37°C. After washing three times in PBST, rat antisera against rTgCSI (1:100 dilution) were added and incubated at 4°C overnight. Cells were then washed three times with PBST, after which goat anti-rat IgG antibody labeled with Cy3 (Beyotime) (1:500 dilution) was added and maintained in darkness for 1 h. After washing three times in PBST, DAPI was added and maintained for 5 min at RT. Next, fluorescent mounting medium (Beyotime) was added and the cells were examined by fluorescent microscopy at 1,000 × magnification (Olympus).

### Effect of rTgCSI protein on proliferation of ANA-1 cells

A cell counting Kit-8 (CCK-8, Beyotime) was utilized to detect the cell viability. Ana-1 cells were seeded in three replicates in 96-well plates (100 μl, 5 × 10^4^/well) overnight, after which they were treated with rTgCSI protein of different concentrations (0, 5, 10, 20, 40, and 80 μg/mL), with pET-32a (20 μg/mL) protein used as control group. Twenty-four hours later, the cells were further incubated with 10 μl CCK-8 solutions for 1 h. Cell proliferation was subsequently calculated based on the OD450 values determined using a microplate spectrophotometer (BioRad Laboratories, Hercules, CA, USA).

### Effects of rTgCSI protein on phagocytosis of murine macrophages *in vitro*

Ana-1 cells were treated with rTgCSI protein at different concentrations (0, 5, 10, 20, 40, and 80 μg/mL) and pET-32a (20 and 80 μg/mL) protein for 24 hr. The cells were then collected and incubated with 1 mg/ml fluorescein isothiocyanate-dextran (FITC-dextran) (Sigma, St. Louis, MO, USA). Next, internalization of FITC-dextran by Ana-1 cells was analyzed by flow cytometry (BD Biosciences, San Jose, CA, USA) as described by Li et al. (2016). Data were analyzed using the Flow Jo 7.6 software (Tree Star, Ashland, OR, USA).

### Effects of rTgCSI protein on apoptosis of Ana-1 cells

Cell apoptosis was estimated using an Annexin V-FITC Kit (Miltenyi Biotec, Germany). After 24 h of treatment with different concentrations (0, 5, 10, 20, 40, and 80 μg/mL) of rTgCSI protein and pET-32a (20 and 80 μg/mL) protein, Ana-1 cells (1 × 10^6^ cells) were harvested and apoptosis was evaluated using a flow cytometer (BD Biosciences, San Jose, CA, USA.) and the Flow Jo7.6 software (Tree Star, Ashland, OR, USA).

### Detection of cytokines

After 24 h of treatment with different concentrations (0, 5, 10, 20, 40, and 80 μg/mL) of rTgCSI protein and pET-32a (20 μg/mL) protein, cell supernatants were harvested. The expression of IL-1β, IL-10, TNF-α, and TGF-β1 of macrophages was then examined using a mouse cytokine ELISA Kit (Boster Biotechnology, Wuhan, Hubei, China). Among them, the detection range of IL-1β was 12.5–800 pg/ml, and that of the other three proteins was 15.6–1,000 pg/ml.

### Measurement of nitric oxide (NO) production

Cell supernatants were collected after 24 h of treatment with different concentrations (0, 5, 10, 20, 40, and 80 μg/mL) of rTgCSI protein and pET-32a (20 μg/mL) protein and analyzed for NO production using a Total Nitric Oxide Assay Kit (Beyotime Biotechnology, Haimen, China).

### Statistical analysis

Data are presented as the means ± SEM. All data obtained from the above experiments were analyzed by one-way analysis of variance (ANOVA) followed by a Tukey test using the Graphpad Prism 5.0 software (GraphPad Software, USA). Differences among groups were considered significant at *p* < 0.05.

## Results

### Cloning, expression, purification, and renaturation of rTgCSI

**Figure 1:**
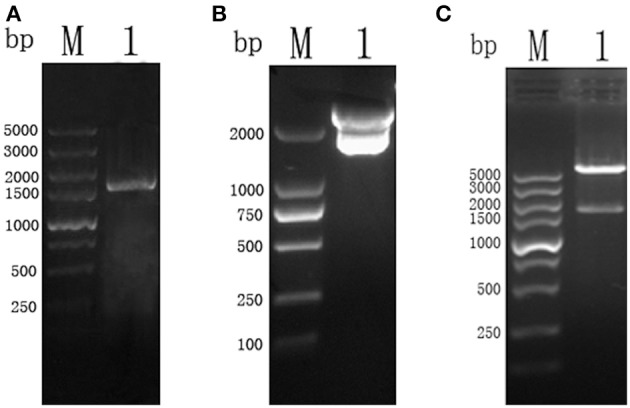
**(A)** PCR results of TgCSI ORF. (Lane M) DNA marker, (Lane 1) ORF of TgCSI. **(B)** (Lane M) DNA marker, (Lane 1), pMD18-T-CSI vector was double digested by *Bam*H I and *Xho* I enzymes. **(C)** (Lane M) DNA marker, (Lane 1), pET-32a-CSI double digested by *Bam*H I and *Xho* I enzymes.

The amplicon of the full length CSI gene of *T. gondii* was successfully purified and cloned into a pMD19-T vector, which was confirmed by restriction enzyme digestion with *Bam*H I and *Xho* I restriction enzymes (Figure 1).

**Figure 2:**
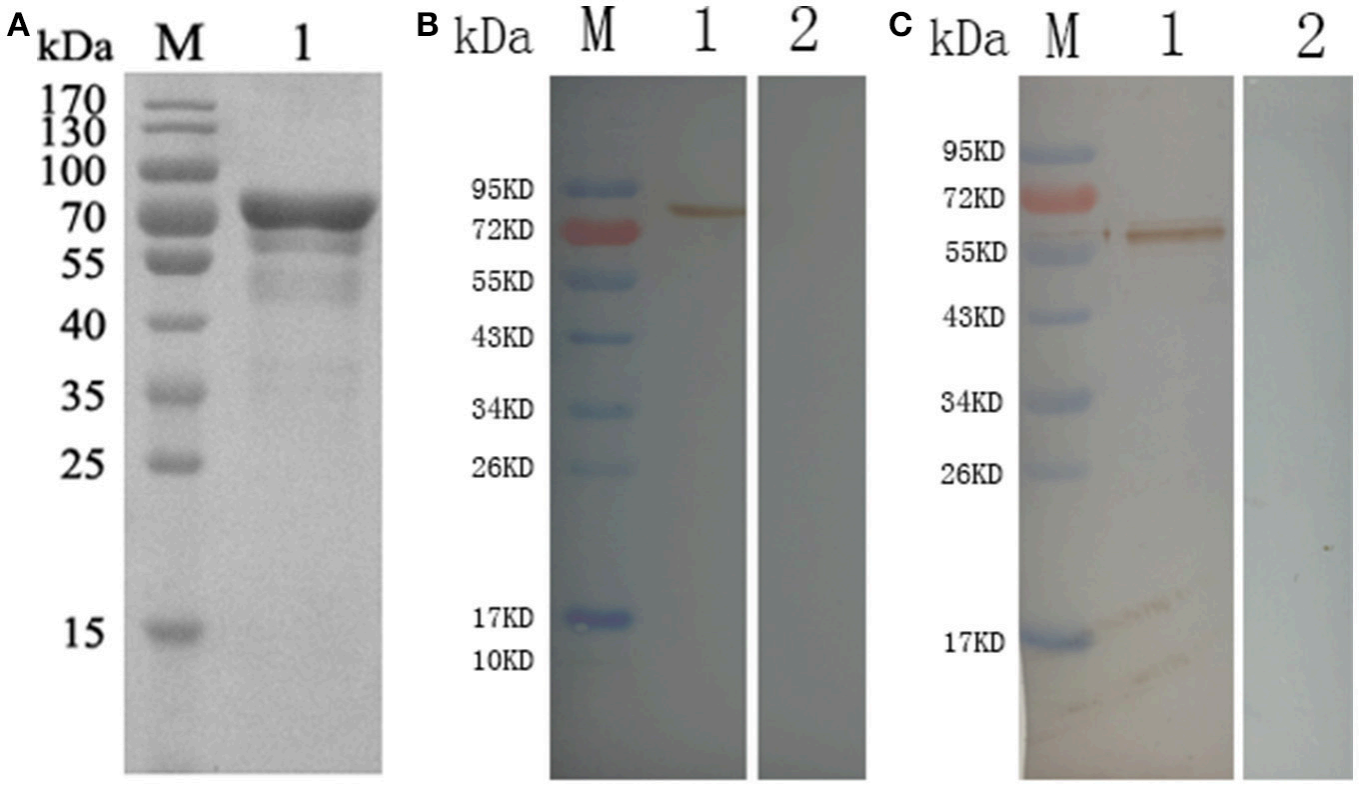
**(A)** Reducing SDS-PAGE of purified rTgCSI. (Lane M) protein marker, (Lane 1) rTgCSI protein. **(B)** Western blot of rTgCSI. (Lane M) protein marker, (Lane 1) recombinant protein TgCSI probed by serum from rats experimentally infected with *T. gondii* as primary antibody, (Lane 2) recombinant protein TgCSI probed by serum of normal rats as primary antibody. **(C)** Western blot of the total soluble protein of *T. gondii* tachyzoites. (Lane M) protein marker, (Lane 1) the total soluble protein of *T. gondii* tachyzoites probed by serum from rats immunized by rTgCSI, (Lane 2) the total soluble protein of *T. gondii* tachyzoites probed by serum of normal rats.

The correct fragment of TgCSI was inserted into the *Bam*H I and *Xho* I sites of a pET32a (+) vector. Reducing SDS-PAGE showed that the recombinant protein was mostly found in the sonicated bacteria inclusion bodies. The molecular weight of native TgCSI was about 60 kDa based on estimation using the DNAstar software. After purification of rTgCSI, a single band of 78 kDa was seen on the SDS-PAGE gel (Figure 2A). The molecular size of the polyhis-tag in the pET-32a vector was about 18 kDa, indicating that the rTgCSI protein was exactly 60 kDa.

### Western blot analysis of TgCSI

To determine if rTgCSI was detected by antibodies against *T. gondii* infection sera, two rats were infected with *T. gondii* tachyzoites. Sera were collected 3 weeks post-infection and used to probe strips of nitrocellulose containing rTgCSI protein (Figure 2B). Recombinant TgCSI protein was recognized as a band of approximately 78 kDa, which is consistent with the recognized molecular weight of the rTgCSI.

To obtain immune serum against rTgCSI, two rats were immunized 5 times with 300 μg rTgCSI. Immune sera collected 7 days after the last immunization were used in western blot analyses to incubate strips of native protein. Controls were incubated with normal rat serum. The results shown in Figure 2C indicate that TgCSI was identified as a weak band of about 60 kDa, which was consistent with the molecular weight of the native protein.

### Protein rTgCSI can bind to murine macrophages (Ana-1)

**Figure 3:**
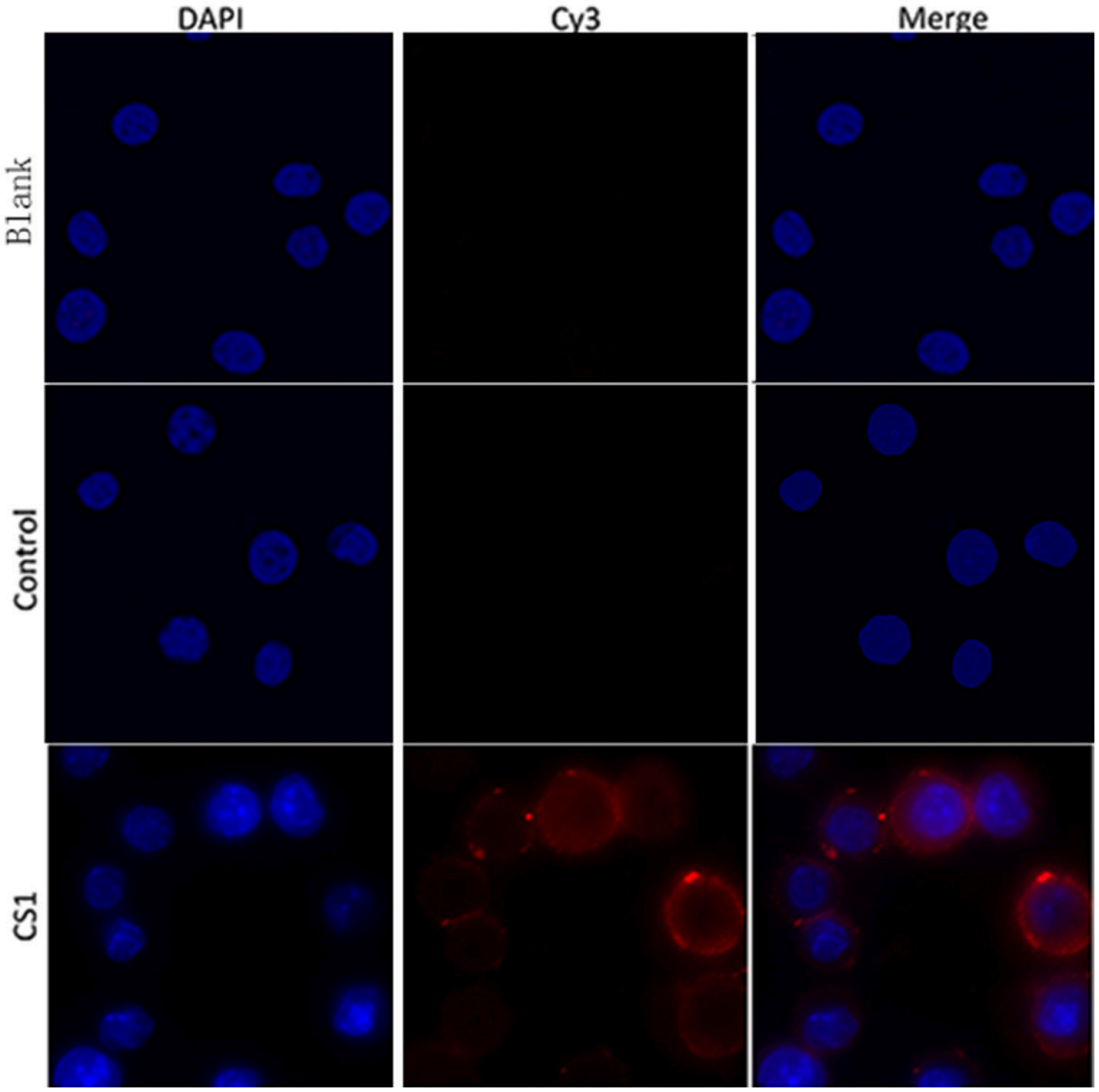
Ability of rTgCSI to bind murine macrophage. Cells were incubated with rTgCSI, maintained with Cy3 labeled goat anti-rat IgG antibody and stained with DAPI. The nuclei of the corresponding cells were visualized by DAPI (blue) staining. Staining of the target proteins (red) was visualized by Cy3-conjugated secondary antibody. Merge, overlap of red and blue channels. No red fluorescence was observed in the control group or the blank group.

To verify whether rTgCSI was a macrophages binding protein, the cells were incubated with rTgCSI. Recombinant protein was labeled with rat-anti-rTgCSI immune serum and visualized with a Cy3 labeled goat anti-rat IgG antibody. Cells were stained with DAPI. As shown in Figure 3, rTgCSI protein bound to Ana-1 cells. The nuclei of the cells showed blue fluorescence staining (DAPI), and the cell surface revealed red fluorescence staining (Cy3) in the confocal microscopy images. In the control group, there was no red fluorescence staining.

### Protein rTgCSI inhibited the proliferation of Ana-1 cells

**Figure 4:**
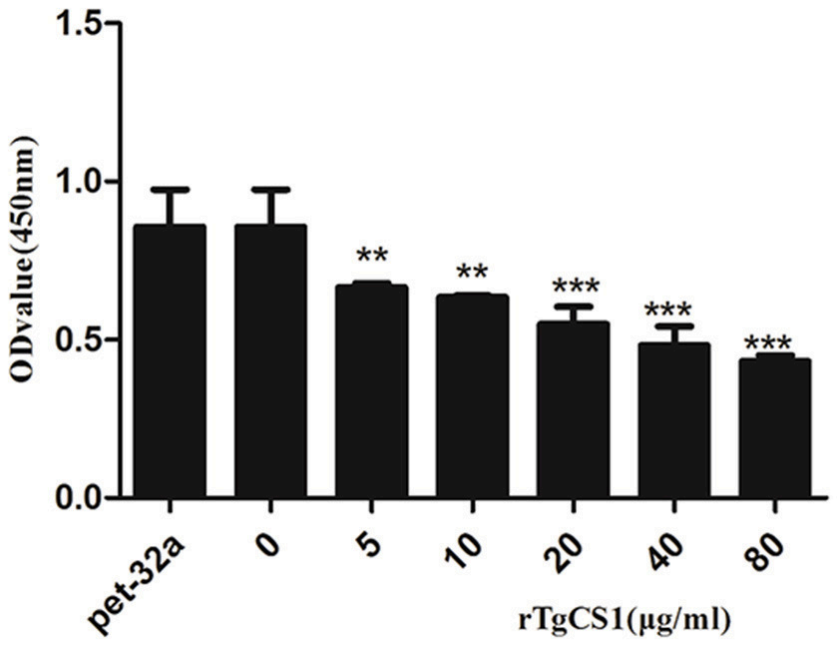
Effects of rTgCSI on cell proliferation of murine macrophages. Macrophages were treated with rTgCSI and incubated with CCK-8. The OD450 value was measured using a microplate spectrophotometer. The cell proliferation index was calculated according to the OD450 value. The data were representative of three independent experiments and the values presented here were the means ± SEM (^**^p < 0.01 and ^***^p < 0.001).

After treatment of Ana-1 cells with different concentrations of rTgCSI and incubation with CCK-8, OD450 values were measured by spectrophotometry. The results showed that there was no significant difference between the group treated with pET-32a protein and the negative control group. The proliferation of groups that received rTgCSI treatment was inhibited significantly in a dose-dependent manner when compared with the control groups (Figure 4).

### Protein rTgCSI showed a dual function on phagocytosis of Ana-1 cells

**Figure 5:**
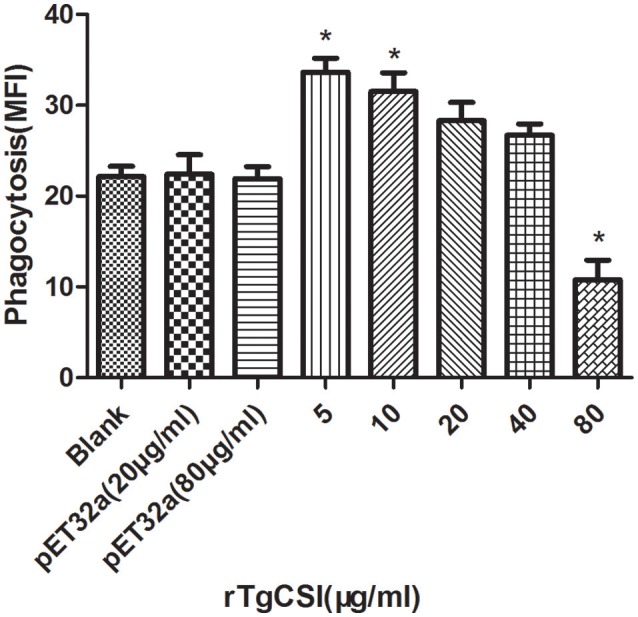
Effects of rTgCSI on cell phagocytosis of murine macrophages. Phagocytosis was measured by flow cytometry after incubation with 1 mg/ml FITC-dextranin PBS at 37°C for 1 h. The cell phagocytosis index was calculated according to statistical data describing the MFI (Median Fluorescence Intensity) value. The data were representative of three independent experiments and the values presented here were the means ± SEM (^*^p < 0.01).

To examine the effects of rTgCSI on macrophage phagocytosis, the FITC-dextran internalization of cells was analyzed by flow cytometry. The results showed that there was no significant difference between the pET-32a protein groups and the negative control group without protein. rTgCSI of low concentration (5 and 10 μg/mL) can enhance the phagocytosis ability of macrophages. Concentrations of 20 and 40 μg/mL had some stimulating effects on the phagocytic ability of macrophages, but these were not significant. However, the 80 μg/mL rTgCSI protein had an inhibitory effect on the phagocytic ability of macrophages (Figure 5).

### rTgCSI protein induced apoptosis in Ana-1 cells

**Figure 6:**
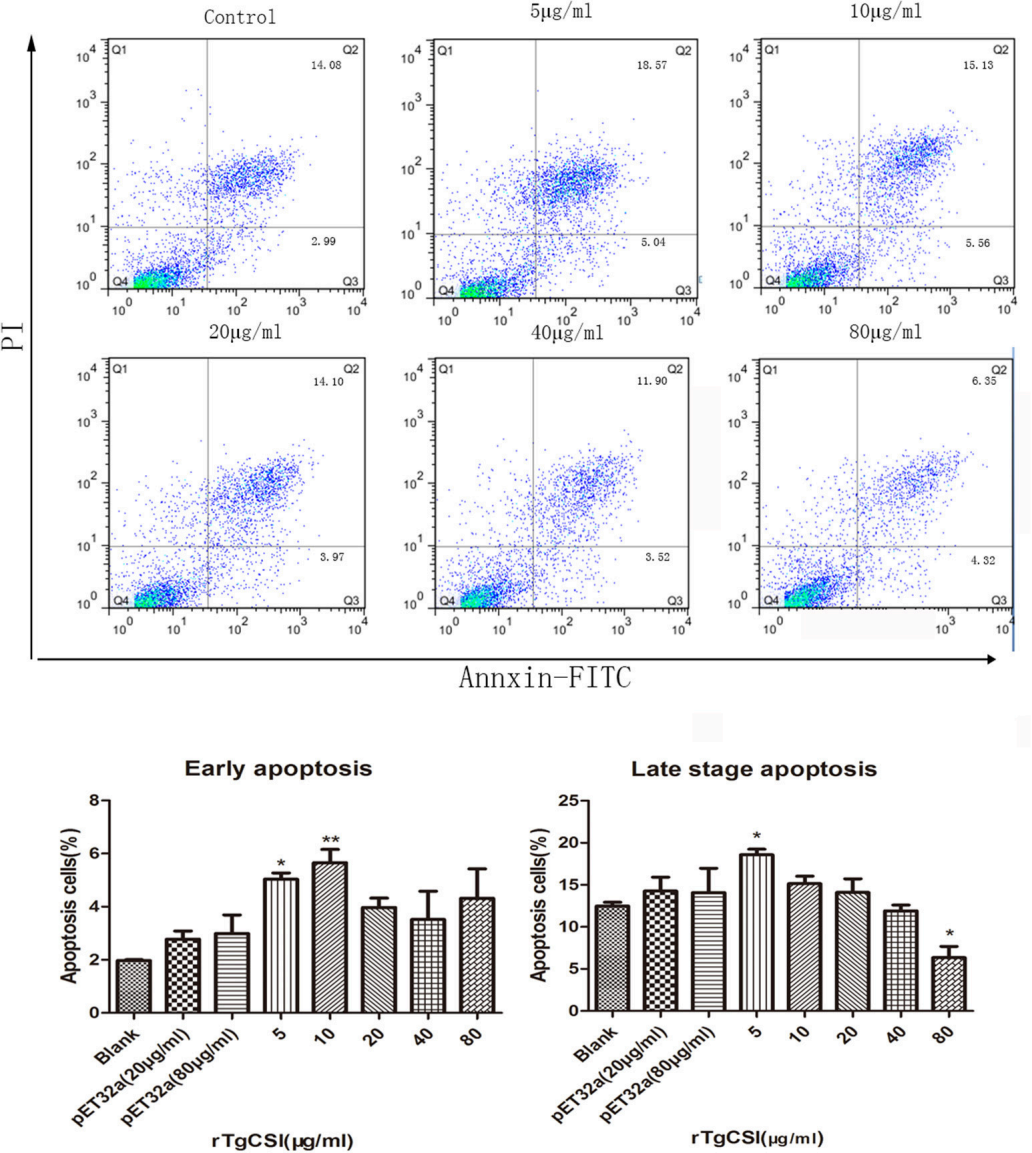
Effects of rTgCSI on the apoptosis of murine macrophages. Cells were incubated with serial concentrations of rTgCSI at 37°C and 5% CO_2_. Apoptosis was measured by flow cytometry after treatment using an Annexin V-FITC kit. The percentage of apoptosis was representative of three independent experiments and the values presented here were the means ± SEM (^*^p < 0.05 and ^**^p < 0.01).

The effects of rTgCSI on the apoptosis of macrophages were estimated using an Annexin V-FITC Kit. As shown in Figure 6, there was no significant difference between the pET-32a protein groups and the blank group. Concentrations of 5 and 10 μg/mL rTgCSI protein significantly induced early apoptosis of Ana-1 cells, whereas 20, 40, and 80 μg/mL had no significant effect on early cell apoptosis. Late stage apoptosis was only induced by 5 μg/mL rTgCSI protein (^*^p < 0.05), while 80 μg/mL rTgCSI protein inhibited the late stage of apoptosis (^*^p < 0.05) of Ana-1 cells.

### Effects of rTgCSI on expression of cytokines in Ana-cells

Mouse cytokine ELISA Kits were used to examine the expression of IL-1β, IL-10, TNF-α, and TGF-β1.

#### rTgCSI promoted expression of anti-inflammatory cytokines

**Figure 7:**
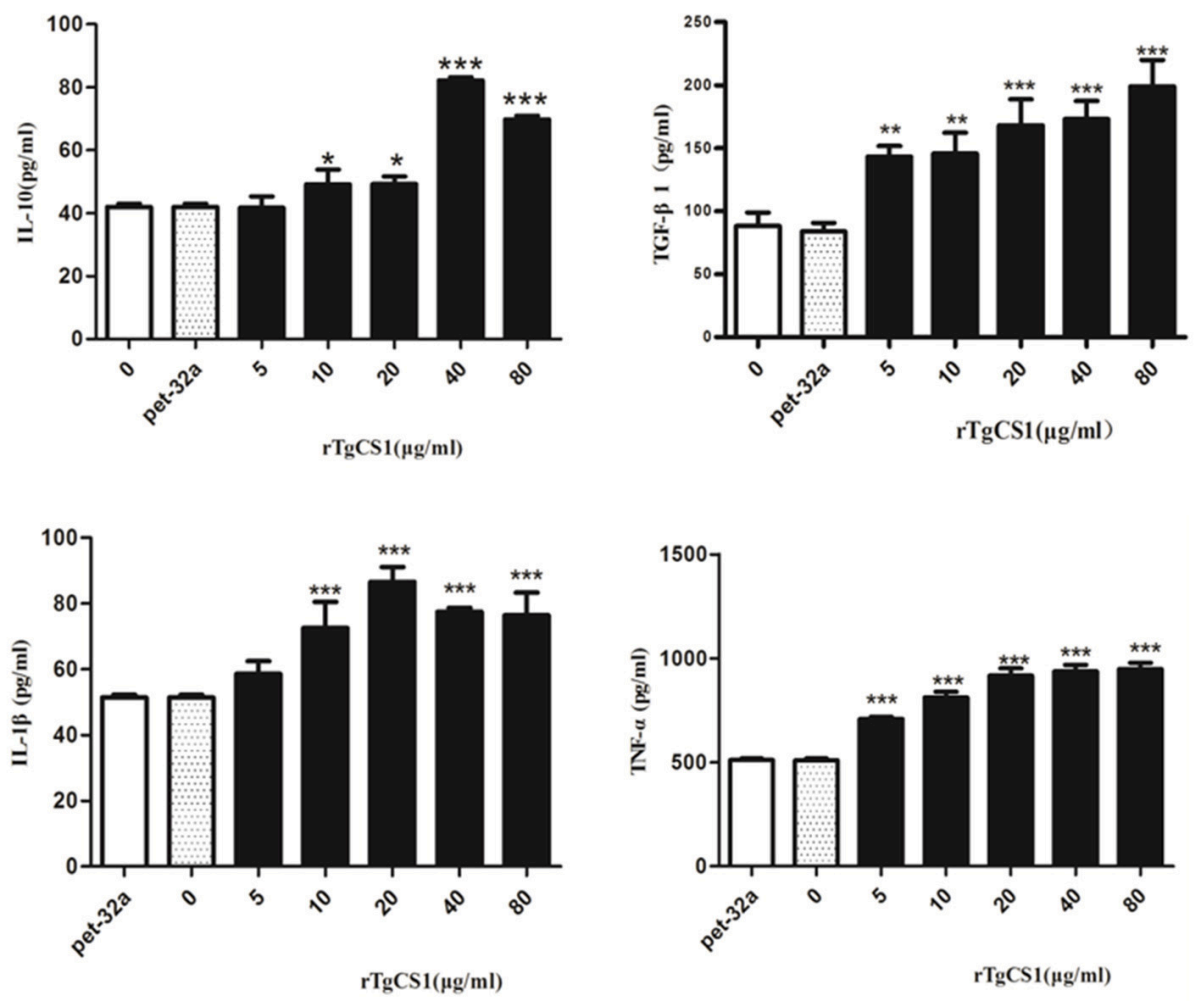
Analysis of the level of multiple cytokine production by murine macrophages *in vitro*. Cytokine secretion in the supernatant of cell cultures was examined according to the mouse cytokine ELISA Kit. The data were representative of three independent experiments and the values presented here were the means ± SEM (^*^p < 0.05, ^**^p < 0.01, and ^***^p < 0.001).

The results of experiments that measured the expression of cytokines showed that there was no significant difference between the pET-32a protein group and the blank group. When compared with the control groups, the production of TGF-β1 in the supernatants of cells receiving rTgCSI treatment was significantly higher and the increased secretion was related to the concentration of rTgCSI (^**^p < 0.01 and ^***^p < 0.001). While 5 μg/mL rTgCSI had no effect on IL-10 production of macrophages, 10 μg/mL and 20 μg/mL rTgCSI could promote the production of IL-10, but the effects were not as obvious at these concentrations as at 40 and 80 μg/mL (^*^p < 0.05 and ^***^p < 0.001 and Figure 7).

#### rTgCSI promoted expression of proinflammatory cytokines

The results showed that there was no significant difference between the pET-32a protein group and the blank group. A significantly higher level of TNF-α was produced in the supernatants of cells treated with rTgCSI (^***^p < 0.001 and Figure 7) compared with the control groups. The production of IL-1β in the supernatants of Ana-1 cells receiving rTgCSI treatment was significantly higher than that of control groups, except for the group that was treated with 5 μg/mL rTgCSI (^***^p < 0.001 and Figure 7).

### Measurement of nitric oxide (NO) production

**Figure 8:**
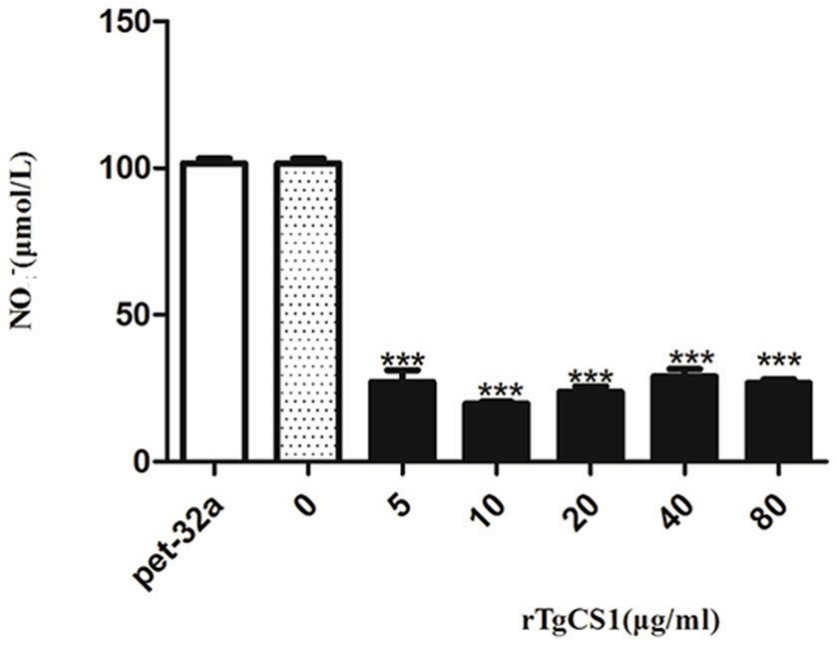
Analysis of the nitric oxide production by murine macrophages *in vitro*. Nitric oxide secretion in the supernatant of cell cultures was performed according to the instructions of a Total Nitric Oxide Assay Kit. The data were representative of three independent experiments and the values presented here were the means ± SEM (^***^p < 0.001).

To examine the effects of rTgCSI on NO production of macrophages, a Total Nitric Oxide Assay Kit was used to measure the NO production in cell supernatants. The results showed that there was no significant difference between the pET-32a protein group and the blank group. However, significantly lower levels of NO were produced in the cell supernatants of groups treated with all concentrations of rTgCSI compared with the control groups (^***^p < 0.001 and Figure 8).

## Discussion

Previous work has shown that when the TCA cycle was chemically inhibited completely, the replication of *T. gondii* was prevented (Macrae et al., 2011). However, exactly how the enzymes of the TCA cycle work in organisms continues to be poorly understood. The first and key enzyme of the cycle is CS, which can catalyze the synthesis of citrate from oxaloacetate and acetyl-CoA (Krebs, 1949), and the rate limiting step of TCA cycle is the reaction in which CSs are involved in Walsh and Koshland (1985). Few researchers studied the function of CSI *T. gondii*. In the present study, we successfully cloned the TgCSI gene, expression of recombinant protein was verified by SDS-PAGE and western blot analyses showed that rTgCSI was immunogenic.

Macrophages are widely considered as critical effectors in hosts for defense against pathogens (Franken et al., 2016). These cells perform both trophic and toxic functions through constitutive and induced endocytosis, phagocytosis, and secretion of various products, including cytokines, growth factors, and metabolites. Their secretory repertoire includes anti-bacterial and proteolytic enzymes, NO, anti-inflammatory cytokines such as IL-10 and TGF-β, and proinflammatory cytokines such as IL-1β and TNF (Gordon and Martinez, 2010).

The primary role of macrophages in response to pathogens is phagocytosis (Weiss and Schaible, 2015); therefore, the ability of phagocytosis can directly reflect the immune functions of macrophages. The work presented in this study suggests that rTgCSI protein has a dual function. Specifically, low concentrations of rTgCSI enhanced phagocytosis, while high concentrations inhibited phagocytosis of macrophages. These findings illustrate that the concentration of rTgCSI plays a decisive role in phagocytosis.

It has been proposed that colony-stimulating factor-1 (CSF-1) regulates the proliferation of macrophages (Pixley and Stanley, 2004). Furthermore, TNF-α was shown to decrease the population doubling time of growth-competent macrophages, as well as that of primary macrophages derived from bone marrow (Guilbert et al., 1993). In this study, although expression of TNF-α was up-regulated, the proliferation of macrophages was inhibited after treatment with rTgCSI. Therefore, the true function of rTgCSI in the regulation of proliferation still needs further research.

Apoptosis has been shown to help in the defense against pathogens. Infection with the obligate intracellular parasite *T. gondii* is well known to trigger host-cell apoptosis (Nishikawa et al., 2007). The activities of many genes influence the cells' likelihood of activating its self-destruction program (Hengartner, 2000). The secretion of NO and other soluble factors released by parasite-infected cells has also been shown to lead to the apoptosis of bystander host cells (Nishikawa et al., 2007). In this study, 5 μg/mL rTgCSI stimulated the apoptosis of macrophages, but 80 μg/mL rTgCSI inhibited the late stage of apoptosis. Therefore, our data suggest that rTgCSI was associated with the regulation of macrophages apoptosis, and that these effects occurred in a concentration dependent fashion.

Macrophages mediate immune response through the secretion of some important immune regulatory factors such as TNF-α, IL-1β, TGF-1β, and IL-10 (Rodrigues et al., 2013; Waters et al., 2013; Berghaus et al., 2014). Barbara reported that *T. gondii* actively interfered with activation of the NF-κB pathway in macrophages, which led to disturbances in IL-12 and TNF-α production, enabling parasite survival within the host (Butcher et al., 2001). Flores (Flores et al., 2008) reported that mice produced significantly less IL-1β and TNF-α during the early phase of infection. Another report provided evidence that TNF produced endogenously during *T. gondii* infection played a role in restraining trophozoite numbers and may prevent death (Johnson, 1992). In the present study, rTgCSI increased the production of IL-1β and TNF-α. These findings suggest that this protein increases the killing effect of macrophages.

Since increased secretion of TGF-β and IL-10 will inhibit the secretion of other cytokines such as IL-2, IFN-γ, TNF-α, IL-4, IL-3, and IL-1, these two cytokines may be critically involved in mediating *T. gondii* induced immunosuppression in the infected host (Ghasemi et al., 2012; Cekanaviciute et al., 2014). In our study, the secretion of IL-10 and TGF-β increased after treatment with rTgCSI; therefore, the ability of macrophages to kill parasites was suppressed and the parasite's proliferation ability was increased. As a result, rTgCSI up-regulated IL-10 and TGF-β secretion and hampered the ability of the host to kill parasites.

It has been proposed that secretion of NO in macrophages can kill and control *T. gondii* multiplication (Adams et al., 1990). It has also been reported that, when infected with *T. gondii*, the production of NO was inhibited in both mouse macrophages (Seabra et al., 2002) and chicken cell lines (Guillermo and Damatta, 2004). In our study, NO production was suppressed significantly after treatment with rTgCSI, suggesting that the rTgCSI protein had a negative effect on NO production and facilitated *T. gondii* infection.

## Conclusion

In this study, we cloned and characterized TgCSI for the first time. Our results indicate that TgCSI is a protein that binds to macrophages. Moreover, rTgCSI protein was shown to have a dual function, with low concentrations enhancing phagocytosis and late stage apoptosis and high concentrations inhibiting phagocytosis and late stage apoptosis of macrophages. The production of IL-10, IL-1β, TNF-α, TGF-β, and early stage of apoptosis of macrophages increased after the cells were incubated with rTgCSI. However, the production of NO and cell proliferation of macrophages were significantly decreased. Considering that the main protective immunity against *T. gondii* is a cellular immune response, rTgCSI had significant effects on the cellular functions of murine macrophages *in vitro*, but the regulatory mechanisms of rTgCSI *in vivo* need to be further investigated.

## Author contributions

XLiu analyzed the experimental data and drafted the manuscript. XSun and QM conducted the study. ML helped analyze the experimental data. ME and MH helped rectify mistakes in grammar. LX, RY, and XSong participated in coordination. XLi conceived of the study and contributed to writing the manuscript. All authors approved the final version of the manuscript.

### Conflict of interest statement

The authors declare that the research was conducted in the absence of any commercial or financial relationships that could be construed as a potential conflict of interest.
